# Pilot evaluation of a second-generation electronic pill box for adherence to Bedaquiline and antiretroviral therapy in drug-resistant TB/HIV co-infected patients in KwaZulu-Natal, South Africa

**DOI:** 10.1186/s12879-018-3080-2

**Published:** 2018-04-11

**Authors:** N. Bionghi, A. Daftary, B. Maharaj, Z. Msibi, K. R. Amico, G. Friedland, C. Orrell, N. Padayatchi, M. R. O’Donnell

**Affiliations:** 1 Columbia University College of Physicians and Surgeons, NY, NY USA; 2 CAPRISA MRC- HIV-TB Pathogenesis and Treatment Research Unit, Durban, South Africa; 3 Division of Pulmonary, Allergy, and Critical Care Medicine, Columbia University Medical Center, NY, NY USA; 4 Department of Epidemiology, Mailman School of Public Health, Columbia University Medical Center, NY, NY USA; 5 McGill International TB Centre, McGill University, Montreal, Canada; 6 University of Michigan School of Public Health, Ann Arbor, MI USA; 7 Yale School of Medicine, New Haven, CT USA; 8 Desmond Tutu HIV Centre, University of Cape Town, Cape Town, South Africa

**Keywords:** Electronic pillbox, Drug-resistant tuberculosis, HIV, Bedaquiline, Real-time monitoring

## Abstract

**Background:**

The introduction of Bedaquiline, the first new antimycobacterial drug in over 40 years, has highlighted the critical importance of medication adherence in drug-resistant tuberculosis (DR-TB) treatment to prevent amplified drug-resistance and derive sustained benefit. Real-time electronic dose monitoring (EDM) accurately measures adherence and allows for titration of adherence support for anti-retroviral therapy (ART). The goal of this study was to evaluate the accuracy and acceptability of a next-generation electronic pillbox (Wisepill RT2000) for Bedaquiline-containing TB regimens.

**Methods:**

Eligible patients were DR-TB/HIV co-infected adults hospitalized for the initiation of Bedaquiline-containing treatment regimens in KwaZulu-Natal, South Africa. A one-way crossover design was used to evaluate levels of adherence and patient acceptance of EDM. Each patient was given a Wisepill device which was filled with ART, Levofloxacin or Bedaquiline over three consecutive weeks. Medication adherence was measured using Wisepill counts, patient-reported seven-day recall, and weekly pill count. An open-ended qualitative questionnaire at the end of the study evaluated participant acceptability of the Wisepill device.

**Results:**

We enrolled 21 DR-TB/HIV co-infected inpatients admitted for the initiation of Bedaquiline from August through September 2016. In aggregate patients were similarly adherent to Bedaquiline (100%) compared to Levofloxacin (100%) and ART (98.9%) by pill count. Wisepill was more sensitive (100%) compared to seven-day recall (0%) in detecting non-adherence events (*p* = 0.02). Patients reported positive experiences with Wisepill and expressed willingness to use the device during a full course of DR-TB treatment. There were no concerns about stigma, confidentiality, or remote monitoring.

**Conclusion:**

In this pilot study patients were highly adherent to Bedaquiline by all adherence measures. However, there was lower adherence to ART by pill count and Wisepill suggesting a possible challenge for adherence with ART. The use of EDM identified significantly more missed doses than seven-day recall. Wisepill was highly acceptable to DR-TB/HIV patients in South Africa, and is a promising modality to support and monitor medication adherence in complex treatment regimens.

## Background

Tuberculosis (TB) is the leading cause of death globally from a single infectious pathogen having surpassed HIV in estimated mortality in 2015 [1]. The emergence and propagation of drug-resistant TB (DR-TB), with an estimated 580,000 new cases in 2015, has led to a marked increase in the number of tuberculosis patients requiring more extensive and complex treatment regimens [2]. Despite the large number of drugs utilized in the management and treatment of DR-TB, current treatments continue to be ineffective in operational settings: success rates range from 48% for MDR-TB to 22% in patients with XDR-TB [3]. Outcomes are often worse for TB patients co-infected with HIV. This has significant implications for management of DR-TB in regions of South Africa such as KwaZulu-Natal, where the majority of DR-TB patients are HIV co-infected [4, 5].

Medication adherence is associated with TB culture conversion in DR-TB/HIV treatment but the full impact of non-adherence and lack of retention in care on treatment outcome is not well understood. Based on biologic understanding and modeling exercises, poor adherence is thought to drive the development of drug resistance in the treatment of *Mycobacterium tuberculosis* (MTB) [6–8]. While considerable attention to measurement of adherence to HIV antiretroviral therapy (i.e. self-report, pill count, pharmacy refills, and electronic monitoring) has produced a robust literature and evidence base, similar work with EDM pillboxes has not been done in TB and TB/HIV co-infection treatment [9, 10]. Real-time monitoring of adherence using a next-generation electronic pillbox (Wisepill) has important potential to identify early adherence lapses prior to TB treatment failure and may have a role to play in the prevention of drug resistance [11]. In addition, electronic adherence monitoring may be beneficial when incorporated within a patient-centered model of improving adherence, that contextualizes missed doses within the clinical, socio-economic, and structural factors surrounding this highly stigmatized group [12].

Bedaquiline is the first new drug licensed for the treatment of DR-TB in over 40 years (Janssen Therapeutics, 2015). In clinical trials Bedaquiline-containing regimens showed an increase rate of 21% TB culture conversion compared with control regimens but MTB resistance to Bedaquiline in the presence of suboptimal drug-levels has already been described [13, 14]. Bedaquiline is dosed intermittently (e.g. three times weekly) and has a long half-life rendering it vulnerable to emergent drug-resistance when medication adherence is sub-optimal. Currently, there are no studies on measurement or improvement of adherence to Bedaquiline in DR-TB/HIV treatment.

We designed this mixed-methods pilot study as preliminary data for a larger study of adherence in DR-TB HIV in South Africa (PRAXIS, NIH R01AI124413). The goals were to measure self-administered medication adherence to Bedaquiline compared to other second-line TB medications and ART in South African patients with DR-TB/HIV coinfection and to assess patient acceptance of a next-generation electronic pill box.

## Methods

### Study site and population

This pilot study was conducted at a TB referral hospital in KwaZulu-Natal, South Africa from September through October 2016. Eligible participants were adults (> age 18) with drug-resistant TB/HIV co-infection on ART who were referred for the initiation of a Bedaquiline-containing treatment regimen. Capacity to give written informed consent in either English or isiZulu was required for enrollment. Participant age, gender, previous TB history, and previous MDR-TB history were obtained by a Zulu-speaking staff member at enrollment in the study and other data was abstracted from the clinical chart.

### Study design

**Fig. 1:**
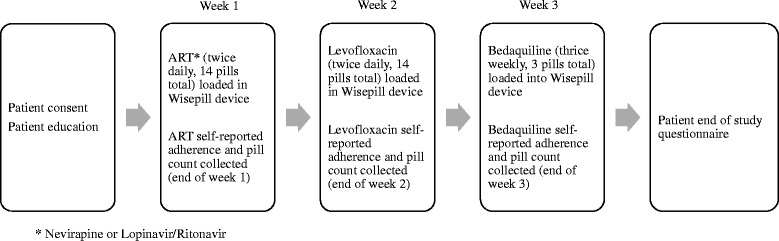
Study design

This study used a mixed methods approach with a prospective one-way crossover design and qualitative patient questionnaires (Fig. 1). On enrollment participants were provided with a Wisepill RT2000 device and instructed on its use. A single type of medication was stored in the Wisepill device at a time to reduce confounding and for practical space reasons. The Wisepill device was sequentially loaded first with ART (either Nevirapine or Lopinavir/Ritonavir, twice daily dosing), then Levofloxacin (twice daily dosing), and lastly Bedaquiline (3 doses per week), each with a one week supply of medication reflecting the dosing. For each week, the patients took the designated medication exclusively from the Wisepill device, without supervision from hospital staff. Other medications in the patients’ treatment regimens were administered by a nurse per the hospital protocols, and nurses were informed that Wisepill medications would be taken by the patients independently. At the end of the week a pill count was performed only for the medication in the Wisepill device, self-reported adherence for the medication in the Wisepill device was recorded based on a brief patient interview, and the next medication was loaded; the process repeated until all three medications were assessed. At the end of the study all patients had a questionnaire administered to elicit opinions about the feasibility and effectiveness of the Wisepill device. During treatment, routine monitoring including electrolytes and electrocardiogram was performed as per the treating clinician.

### Wisepill adherence monitoring device

**Fig. 2:**
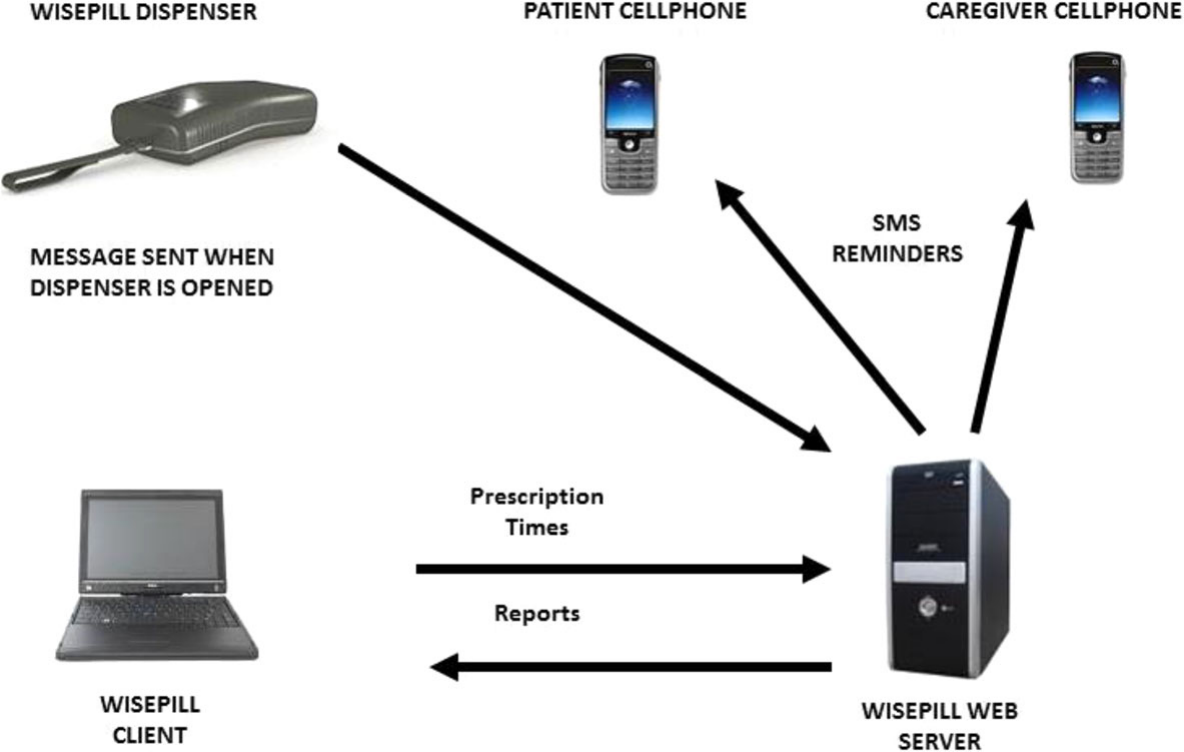
Wisepill device functions. The Wisepill device transmits a wireless signal to the Wisepill web server when device is opened. Device openings are recorded in real-time on the online dashboard and available for users to view. If the SMS reminder service is used, the patients/caregivers may receive message reminders when a medication dose is due or overdue. Image obtained from Wisepill Technologies with permission

**Fig. 3:**
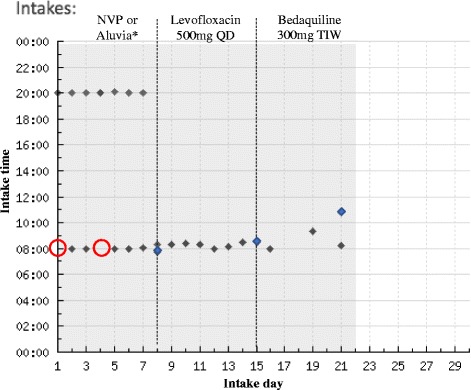
Example of participant intakes reported on Wisepill online dashboard. Example online dashboard display of device openings for a single participant during the three-week study period. The first day of the next medication week is shown with a dotted line. Medication dosing was self-administered. Blue diamonds indicate extra Wisepill device openings for weekly pill count or reloading medications. Red circles indicate a missed expected Wisepill device opening. *Nevirapine (NVP): 200 mg BID; Aluvia: Lopinavir 200 mg – 50 mg Ritonavir BID

The Wisepill RT2000 3G (‘Wisepill’) device has dimensions of 30 × 60 × 130 mm and includes a seven-compartment insert. It is powered by a 3.7 V lithium polymer rechargeable battery and utilizes a Subscriber Identity Module (SIM) card and GSM cellular telephone network. Each time the device is opened, a cellular signal is transmitted to and recorded on an internet server based in Cape Town, South Africa (Fig. 2). Research staff can access this data immediately by signing into the secure Wisepill Web Server **(**Fig. 3). In the case of device power failure or signal transmission malfunction, the Wisepill device is capable of storing data for later transmission when connectivity is re-established. As part of this study the SMS messaging functionality was not used.

### Adherence data and measures

Adherence was measured via three methods: self-reported, weekly pill count, and Wisepill data. A staff member not affiliated with the treatment team obtained patient self-reported adherence at the end of each week by asking in the patient’s language: “How many doses of the medication did you miss?” Weekly pill count was ascertained at the end of each week of medication by counting the number of pills remaining in the pillbox before loading the next week of pills. Wisepill data was collected continuously over the three weeks and downloaded from the online Wisepill Web Server at the end of the study. Adherence was determined utilizing the following formula for each week of medication: (number of doses taken)/(number of prescribed doses). Adherence levels were determined and compared for all three measures.

### Acceptability and feasibility of the Wisepill device

A structured, open-ended qualitative questionnaire was administered by an isiZulu-speaking staff member on the last day of the three-week study period to document participant perspectives and determine feasibility and acceptability of the Wisepill device. Questions investigated the participants’ experiences with and views on using the device and real-time monitoring, preferences regarding medication in the pillbox, ease or difficulty of use, storage of the device, potential challenges with stigma while using the device, and recommendations for improvement.

### Data analysis

Descriptive statistics including medians, interquartile ranges, and frequencies were calculated. Correlations of measures of adherence were calculated using paired sample t-tests and tests of significance were performed using Fisher’s exact tests (SPSS Version 24). Sensitivity and specificity were calculated using standard methods and confidence intervals were calculated using Clopper-Pearson methods. Data from the open-ended study questionnaires was translated into English, checked for accuracy, and subjected to qualitative content analysis [15].

## Results

### Participants

There were 21 inpatients enrolled in the pilot study (Table 1). The participants were TB/HIV co-infected patients on anti-retroviral therapy (either Nevirapine or Lopinavir/Ritonavir, both twice daily dosing) and a TB treatment regimen that included Levofloxacin (once daily) and Bedaquiline (three times weekly). The median age of the participants was 42 years (IQR 33.5–54); 12 of the 21 were men, and 6 (29%) reported previously being diagnosed with TB. Six patients with a history of TB reported having completed their prior treatments. Only one patient reported having a previous episode of MDR-TB. One patient exited the study after completing the week of ART.

**Table 1:** Participant characteristics

Characteristics	Participants
N	21
Median age, years (IQR)	42 (33.5–54)
Gender, n (%)	
Male	12 (57)
Female	9 (43)
HIV status, n (%)	
Positive	21 (100)
ARV, n (%)	
Yes	21 (100)
Previous TB, n (%)	
Yes	6 (29)
No	13 (62)
Not known	2 (9)
Previous MDR-TB, n (%)	
Yes	1 (5)
No	18 (86)
Not known	2 (9)

### Adherence measures

For the 21 patients, there were 489 possible adherence events (i.e. total expected doses of medication in the time period to achieve perfect adherence). Pill count detected 3 missed doses total among the patients. Using pill count as the standard, patient-reported seven-day recall detected 0/3 missed doses detected by pill count whereas the Wisepill device identified all missed doses (3/3), with a significant difference between self-reported and Wisepill measures of ART adherence (*p* = 0.02). All 3 missed doses occurred with ART. There were no non-adherence events reported by pill count for Levofloxacin or Bedaquiline. The difference between pill count adherence for ART and anti-TB medications was not statistically significant (*p* = 0.602).

Utilizing pill count as the standard measure of adherence, the Wisepill device had a sensitivity of 100% (95% CI: 29.2–100%) and a specificity of 98.8% (97.3–99.6%). Interviewer-acquired self-report of doses missed over the past week as a measure of non-adherence had a sensitivity of 0% (0–70.8%) and a specificity of 100% (99.2–100%) (Table 2).

**Table 2:** Performance of adherence measures detecting missed doses: Wisepill versus seven-day recall, pill count as standard. (*N* = 21)*

	Self-reported adherence	Wisepill adherence
Sensitivity (95% CI)	0% (0–70.8%)	100% (29.2–100%)
Specificity	100% (99.2–100%)	98.8% (97.3–99.6%)

*21 patients with 489 possible adherence events. For Wisepill: 3 true positives, 6 false positives, 0 false negatives, 480 true negatives. For self-report: 0 true positives, 0 false positives, 3 false negatives, 486 true negatives

The Wisepill device also reported a greater number of missed doses (represented by the number of times the device was opened) than was identified by pill count: 9 missed doses (1.8%) of the 489 total expected doses, with three of these doses reflecting missed doses captured by pill count as well. Eight of the missed doses were identified during the week of ART and one of these missed doses was noted during the week of Levofloxacin. There were no non-adherence events reported with Bedaquiline using any method of measuring adherence, the highest adherence among the three medications in this study.

### Acceptability and feasibility for electronic device (Table 3)

All participants of the pilot study reported they had a positive experience using the electronic device over three weeks. Participants felt the device had helped them to organize their medications, and to keep their medications safe.

**Table 3:** Wisepill acceptability and experiences: themes identified from end-of-study questionnaire on device use and adherence monitoring

	*N* = 20*	Percent**
Ease of using Wisepill		
Easy to use	19	95%
Not easy to use	1	5%
Prefer to use Wisepill for medications	19	95%
Medication(s) easiest to take with Wisepill		
ART	14	70%
Levofloxacin	2	10%
Bedaquiline	10	50%
Challenges of using Wisepill		
Difficulty opening the box	3	15%
Required extra demonstration of how to use device	1	5%
Worry that children will find/use the device	2	10%
Fear of missing a dose	1	5%
Worry about losing the box during travel	1	5%
Disconcerted by the flashing light	1	5%
Loss of confidentiality regarding disease status	0	0%
Benefits of using Wisepill		
Maintains confidentiality regarding disease status	6	30%
Keeps medications safe	9	45%
Keeps medications organized	3	15%
Perception of being monitored		
Motivates to take medications	9	45%
Induces sense of being cared for	7	35%
Popularity of Wisepill device		
Fellow patients liked the device	11	55%
Fellow patients disliked the loss of autonomy	1	5%
Fellow patients fear repercussions of missing dose	1	5%
Family would react positively to the device	18	90%
Family would be curious about the device	1	5%
Recommendations		
Increase size of pillbox to accommodate more medications	10	50%
Alarm/reminder to take medications	5	25%
Device should have a color	1	5%
Device should only be given to dedicated people	1	5%

*20 of the 21 enrolled patients completed an end-of-study questionnaire

**Patients expressed multiple views; percent reflects (number of patients)/(total number of patients) for each statement independently

It stores my medication nicely, and I can have my medication nicely packed so that not everyone can have access to it.

Acceptability toward the Wisepill device was strongly facilitated by its ease of use. The majority of participants encountered no difficulties removing the medications from the device every day. However, a small number of participants with stiff joints experienced some difficulty opening the device and one participant required an extra demonstration to master handling the device.

### Remote monitoring

Participants understood that they were being remotely monitored by the electronic device, and most had no issue with this monitoring. Many of them viewed Wisepill as an indication that the health care workers/health system cared about their health and treatment success, as reflected in the following quotes.

It made me feel very important to have people worried about how I take my medication.

I had no problem with [monitoring], it’s like driving on a road where there are traffic cops, I understand that they are there to control and to make sure that people are safe on our roads.

This form of monitoring encouraged them to take their medications at the prescribed times, as explained by one patient:

I do not have a problem because I am a person who can take medication without supervision. But being monitored also motivated me to make sure that I take medication.

However, a few participants were worried about failing to adhere to the specified dosing time: “if you miss the correct time you become very scared.”

### Public use

Participants were asked about how their use of the device may be perceived by others around them. No participant felt the device would invite stigma or a loss of confidentiality about their HIV or TB status. Indeed, several patients believed the device helped to maintain their confidentiality.

Yes I would love to use [the device] especially because people undermine and discriminate you when you are on ARVs, so the device is very helpful because nobody can see what is inside.

Most participants also believed that their fellow patients “liked” or “wished they were also given” an electronic device, and nearly all of them were confident that their family and friends – even if inquisitive – would accept them using this device when they were discharged home.

A few participants acknowledged that the device was likely to cause some curiosity at home, particularly because of the flashing light, and were concerned if it were to be discovered and used by their children, or altogether misplaced during travel.

### Preferences

We asked participants about their personal preferences for Wisepill medications. Most indicated that Wisepill was easiest and most convenient for ARVs because they were already accustomed to taking these medications independently, without a nurse’s supervision.

I enjoyed using [Wisepill] more when ARVs were loaded because they are the only of medications I take at my own time. Unlike other meds which I must take together with what is going to be administered by the nurse.

Among the anti-TB drugs, patients preferred to pack Bedaquiline into the Wisepill device, because they felt it was conducive to the drug’s unique thrice weekly dosing; all other medications were taken daily. Time in the study was also a factor; several patients felt the longer they used it, the more comfortable they became using the device.

When participants were probed about their suggestions for future use, many of them requested increasing the size of the device to hold more medications. Other popular suggestions included adding an alarm reminder for taking medication. One participant suggested such devices should be reserved for “dedicated people, not people who will open it and throw away the tablets.”

## Discussion

This pilot study of medication adherence in DR-TB/HIV co-infected patients demonstrates that use of real-time electronic adherence monitoring is effective in capturing non-adherence events and is highly acceptable to patients. In this study, the Wisepill RT2000 device provided real-time feedback on adherence to the online server that was highly correlated with pill count and superior to self-report at capturing non-adherence events when using pill count as a reference standard. Given that Wisepill measures suggested a greater number of missed doses than self-report as well as pill count, electronic adherence monitoring may capture more non-adherence events and its superiority and efficacy to measure adherence and guide adherence support interventions for DR-TB/HIV treatment should be evaluated in future studies.

Electronic device monitoring was able to capture adherence data equally for Bedaquiline, Levofloxacin, and ART suggesting that it is capable of effective monitoring for TB medications as well as the HIV medications, for which electronic adherence monitoring has been previously studied [16–20]. In this study, there were more non-adherence events reported by pill count for ART than for Bedaquiline (not statistically significant), contrary to a preceding study indicating that patients are less likely to adhere to DR-TB treatments compared to ART [21]. This may be because patients in this study were recently switched from Efavirenz, a well-tolerated, once-daily anti-retroviral drug to Nevirapine, a less tolerated, twice-daily drug in order to initiate Bedaquiline therapy. High adherence to Bedaquiline may also reflect better tolerance to Bedaquiline as compared to older second-line TB medications.

The use of an electronic device for dispensing HIV or DR-TB medications was a positive experience for all patients in this study and there were few technical issues reported. Several studies of HIV treatment have demonstrated the acceptability of Wisepill devices; in support of the current literature, this pilot study shows that monitoring was not a concern for patients and this level of supervision was viewed as a sign of caring or a means of motivation [17–19]. Participants in this study felt that keeping the pills in an unmarked electronic device protected confidentiality and reduced the potential for stigma. This suggests that the use of Wisepill might be more acceptable and perhaps even preferable when patients are concerned about the stigma of their diagnosis. This study did not explore the use of adherence reminder messages, but given that several participants felt an adherence reminder function may be useful, this function should be evaluated in future studies. In general, patients expressed a willingness to use the device during a full course of DR-TB treatment, which has yet to be studied on a larger scale.

This study was limited by the short duration of follow-up on the patients and potential biases introduced by the order of medications by week. The temporal bias may have affected adherence to ART (the first week) as compared to other medications, though this is less concerning given the high acceptability and ease of use regardless of medication reported in qualitative assessment. In addition, the small sample size and hospital inpatient monitoring may limit generalizability to the outpatient setting. Other limitations include incomplete qualitative assessment and possible confounding due to unmeasured variables, such as education. For this pilot study, qualitative data was limited to descriptive content analysis; contextual factors such as education and role of the clinic environment were not fully explored. We plan to address these limitations in a future larger study (PRAXIS, NIH R01AI124413).

There are a variety of electronic health devices used in studies for adherence monitoring. The Wisepill RT2000 device used in this study is one of several Wisepill devices available. The Wisepill evriMED500 and evriMED1000 are table top dispensers with USB only or 2G network access to data, respectively. They are also loaded with 5–10 different medications at a time. The characteristics and goals of this pilot study supported the use of the Wisepill RT2000 device for evaluation of adherence of one medication at a time with a smaller device.

Electronic health interventions, including mobile health, have been used in supporting TB treatment adherence with mixed results. In the largest randomized controlled trial to date (*N* = 4173), comparison of 4 study arms (1 control, 3 intervention) showed that the use of text messages as reminders for TB medication adherence was not effective in decreasing missed doses (*p* = 0.649), but electronic medication packaging (with recorded pillbox openings) succeeded in improving TB medication adherence (*p* = 0.008) [22]. Next-generation cloud-based electronic monitoring devices that transmit pill box openings in real-time, on the other hand, have not been used in TB treatment adherence support. In HIV treatment, next-generation electronic pillboxes for adherence monitoring have been used in low and middle-income settings. A study in the U.S. reports similar adherence to HIV ART when using an electronic adherence device or self-report to measure adherence [19]. On the other hand, in a randomized control trial in Botswana, electronic adherence monitoring devices for HIV ART recorded lower adherence rates than self-report and were also shown to be feasible in a low-income setting [20]. Similar results were seen in a rural African setting using a Wisepill device, where high acceptability of the device was also noted [18]. This study contributes to the current knowledge base by applying the use of a Wisepill device to DR-TB drug regimens in HIV/AIDS co-infected patients.

The results of this pilot study are encouraging for the use of electronic monitoring adherence in DR-TB/HIV drug regimens. These results will inform a larger study on the effect of electronic monitoring on adherence, feasibility, and acceptability of this device for second-line TB medications. With increasing use of Bedaquiline for the treatment of DR-TB, further studies on adherence to TB regimens will be required and essential for protecting the efficacy of this new and long-awaited drug.

## Conclusions

The Wisepill device shows promise in providing real-time adherence information in a way that is effective and acceptable to the patient. It has the potential to identify lapses in adherence and possibly improve adherence with real-time intervention before these lapses can lead to a negative impact on treatment success. Self-report and pill count depend on patients continuing to attend clinic visits, do not provide data on potential non-adherence in real-time, may be inferior to Wisepill in capturing non-adherence (self-report), and may not provide the reported confidentiality of the Wisepill device. Thus, the use of electronic monitoring devices appears to be applicable to DR-TB treatment in addition to ART, and may have significant advantages to other commonly utilized adherence monitoring methods.
